# Notes and News

**Published:** 1889-07-13

**Authors:** 


### Notes and News.

Collected and Communicated.

With the exception of the autopsy room and the laundry, all the buildings of the Johns Hopkins Hospital, Baltimore, are connected with a system of covered corridors. Some of these are 500 feet long, and altogether would exceed 1,200 feet in length. These afford excellent walks for the exercises of convalescent patients. Even in winter they can be used, as all of them are moderately warmed by the steam-coils.

Dr. Edward Seaton's lecture at the Sanitary Institute on "The Infectious Hospitals of London as a Defence against Epidemics" was well attended. Dr. Seaton put the accommodation at 4,000, and praised the organisation, including the ambulance system, for its completeness. He recommended the establishment in the metropolis of a hospital rendered innocuous by passing the air coming from it through fire. He advocated compulsory notification of infectious disease in London.

The annual meeting of subscribers to the Liverpool Convalescent Institution at Woolton took place on June 29th, and was well attended, many visitors enjoying a stroll in the beautiful grounds and the hospitality of the matron, Miss Smith, whose name is still constantly on the tongues of her Greenwich friends. The report was read by the secretary, which set forth that during the past year very few events had transpired in connection with the institution to call for special remark. In the height of summer the institution was full, and the total number of patients received during the year had been 100 more than in any previous year. The terms of admission were at the commencement of the past year reduced from 10s. to 7s. 6d. per week; and probably to this cause, more than to any other, was to be attributed the increase of numbers. Although the reduction of fees had undoubtedly resulted in the increased usefulness of the institution, the change had nevertheless sensibly affected its finance, for at its close there was a balance of over £285 due to the bank, notwithstanding that the year commenced with a balance of £50 to its credit.

Bromsgrove, near Birmingham, is now to have its cottage hospital. An enthusiastic meeting was held last week, and large sums promised. Dr. Chavasse offered to build and furnish a male ward for six beds, to be called the Arthur Ryland ward. Lord Windsor also promises £100, and Mr. Corbett, M.P., £500. At this rate the hospital will soon be built, and we hope will be a credit to the promoters of the scheme.

Lady Idina Brassey lately presented a large number of certificates to successful ambulance pupils at Hastings. Mr. Julius, one of the lecturers, said his labour had been one of pure love, for he had had the greatest pleasure in helping. One thing which he thought fully repaid one for what he had done, was the two cases which had been brought before their notice that day. In one case, a lady (the student referred is Miss Emily Welch) restored another person who had been in the water a long time, and who was exhausted, and would probably have died but for her aid; whilst the other was that of a fractured thigh, Mrs. Nelson, of Queen's-road, putting up the fracture. The case was sent to the hospital, and the doctor there congratulated the lady on her labour. Dr. Marshall was presented with an inkstand and a cigarette-case by his ambulance pupils.

The second annual meeting of the friends and supporters of the London Skin Hospital was held on Tuesday at the Whitehall Rooms, Hotel Metropole, the Marquis of Carmarthen, M.P., in the chair. The hospital, as is well known, was established in 1887, and since then there have been 2,000 patients relieved, and the attendances exceed 10,000. The number admitted during the past year was 975 new out-patients, and the attendances numbered 5,407, as compared with 725 patients and 3,398 attendances in 1887-8. Notwithstanding the utmost economy, the expenditure during the year has exceeded the receipts by £107. The existing liabilities now amount in the aggregate to £497, and the chairman earnestly appealed to the friends of the society and the benevolent public for funds. Reference was made to the recent death of Father Damien, and also to the case of leprosy in this country referred to by H.R.H. the Prince of Wales, and Dr. G. B. Concanon pointed out that this is a particularly appropriate time for giving the most hearty support to this estimable institution. On the motion of Dr. James Startin, the new rules which have been drawn up were unanimously approved. The hospital is situated at 47, Cranbourne-street, Leicester-square.

The annual meeting of friends of St. Helens Hospital, Lancashire, has been held, the Mayor presiding. The report stated that the hospital continued to maintain its previously good position, and the committee had been enabled during the past year to thoroughly cleanse and paint it throughout, and also to renew the linen and other necessaries, so that in the matter of equipment it would now compare favourably with any kindred institution in the country. The penny-a-week subscriptions continued to be steadily maintained at last year's very satisfactory figures, and the committee had decided to send patients who subscribed a penny a week, free of charge, to the Devonshire Hospital, Buxton, and the Eye and Ear Infirmary, Liverpool, in addition to the Convalescent Hospital at Southport, as heretofore. The number of patients admitted during 1888 was 225, against 176 in 1887, and 140 in 1886, and the total number of days were 8,439 in 1888, 8,072 in 1887, and 5,588 in 1886. The receipts showed a balance of £473 15s. 10d. from last year; annual subscriptions and donations, £256 7s. 6d.; weekly subscriptions at works, £729 12s. 11d.; collections at places of worship, £122 3s. 10d.; and cash from other sources made a total revenue of £1,700 8s. 7d. There was a balance in hand £492 12s. 9½d.

The following vacancies are announced this week, the amount of the salary in each case being given after the name of the institution :

**Resident Medical Officers.**

Hull Royal Infirmary. Junior Assistant House Surgeon.—£50, board, &c.
Norfolk Hospital. Assistant to House Surgeon.—Board, &c.
Clayton Hospital, Wakefield. House Surgeon. — £120, residence, &c.
Northumberland County Asylum. Clinical Assistant.—Board, &c.
Carlisle Infirmary. House Surgeon.—£70, board, &c.
Royal Berks Hospital. Assistant House Surgeon.—£40, board, &c.
North Staffordshire Infirmary. Assistant House Surgeon. Board, &c.

---

Lieut. H.R.H. Prince George of Wales, K.G., has consented to lay the foundation-stone of a new branch hospital of the Seamen's Hospital Society, at the Royal Victoria and Albert Docks, on Monday next, at four o'clock precisely.

---

Our readers will please note that an excellent musical matinée will be given at the Steinway Hall on Wednesday, July 17th, at three o'clock, in aid of the Central London Ophthalmic Hospital. A large number of distinguished patrons of the hospital will be present, and several eminent artistes have kindly lent their services. Those who wish to spend a pleasant afternoon, and benefit a deserving charity at the same time, had better apply at once for tickets at the Central London Ophthalmic Hospital, 238, Gray's-inn-road.

---

There is a very pretty little hospital at Llandrindod, in Radnorshire, over which Miss Jeffrey presides as matron. Llandrindod is likely to become famous as a health resort soon, now that we are beginning to appreciate our own mineral springs; but locally it has been famous for many years. It is situated on a breezy common 700ft. above the sea-level, and the air is very bracing, and the waters have a decided tonic effect No wonder that the applicants are more numerous than the beds in the hospital, and that more than half the patients come from other counties. Only under those circumstances other counties ought to support the hospital, for it is sadly in need of funds.

---

The Physical Development Society, which has its head-quarters at the Polytechnic, is doing its best for the health of the young men of the day. The subscription is only six-pence a quarter, and every member has presented to him a card telling him what to avoid and what to do. Very sensible is the advice offered : to wear wool next the skin ; to take a daily tub ; to walk as much as possible ; to avoid hot rooms and the habit of taking small quantities of alcohol, etc. The president of the society is Dr. G. W. Hambleton, who is now trying to start a Cottage Hospital at Shanklin for the cure of consumption ; and the vice-president is Mr. J. E. K. Studd, one of the well-known family of cricketers.

---

The Medical and Physical Society in connection with St. Thomas's Hospital gave a pleasant conversazione on June 28. The visitors were numerous, and there was plenty for them to look at. Specimens of medical works of the 15th and 16th centuries, and the commonplace book of John Ward, who was vicar of Stratford-on-Avon in Shakespeare's lifetime. This curious book contains the only known reference to the cause of Shakespeare's death. Sir H. Doulton lent a potter's wheel and some art pottery. There were medical caricatures by Cruikshank and Bunbury, and there was a fine display of instruments and batteries, with the latest inventions for electrical illumination. There was a good band in attendance, and everything proved highly successful.

---

At the fifty-first annual meeting of Great Yarmouth Hospital supporters on June 27, it was agreed that in future ministers should be given admission tickets in return for collections made. The in-patients numbered 300 during the year, and the out-patients 3,430. The accountant stated that the annual expense was now £2,000 a year, and their income £1,600. The building fund receipts, including donations and legacies, had amounted to £10,162 10s. 7d., all of which had been expended, and £22 5s. 1d. was due to the treasurer.

---

It is greatly to be regretted that a quarrel should have arisen at St. Albans about the hospital demonstration, and that so much choler should have been exhibited. Lately the demonstrations have produced but a slight pecuniary result, and it was only natural the organisers should have sought for some change which would arouse interest and add to the funds. It was quite unnecessary to meet the proposal of a Wednesday evening service with abuse, and we hope all will shortly agree to arrange such a service, and carry it out with enthusiasm and goodwill.

---

The all-year weekly collection scheme has been adopted by the Liverpool Hospital Saturday Fund, the following being their reasons for so doing : (1) That, as a matter of fact, the all-year boxes have been in the past far more productive than the annual, the former yielding last year £2,048, against £855 in the latter. (2) That, as a general rule, it is always safe for a charity to depend on a regular and sustained habit of contribution than on a single effort. (3) That this habit is of double importance when the income of the contributors is limited and comes in weekly. (4) That the method of all-year collection creates the very habit on which its success depends, and is in itself an education in charity. (5) That it would create and foster a practical interest in the hospitals throughout the whole year among the working men of Liverpool. (6) That if this method was thoroughly organised and established, the Hospital Saturday Fund would equal and exceed the Hospital Sunday, and the hospitals of the city, so valuable to working men, and so dependent on their support, would be relieved from the danger of disastrous reduction. Liverpool is rather hurt because their fund only reaches £3,000 against £5,000 collected at Leeds.

---

It was a pity that the prize-giving at Charing Cross and the garden party at the Cancer Hospital both coincided with the Shah's visit to the City. Many people were afraid to venture to Charing Cross in case of a crowd, but Lord De L'Isle and Dudley turned up all right, and took the chair. In the report read by the sub-dean it was stated that, notwithstanding the competition between the medical schools of the metropolis and the attractions of universities, Charing Cross Medical School still held its own. Seventy students had entered during the session, the total number in attendance being 221. The report added, moreover, that plans for the enlargement of the school had been adopted, and would shortly be carried out. The prizes, etc., were then distributed, the most successful students being F. H. A. Taylor, C. H. J. Lockyer, T. E. Constant, A. H. Smith, A. W. W. Hoffman, N. R. J. Rainier, G. H. Hooper, J. H. Day, W. R. Barrett, H. S. Baker, J. Bushfield, G. F. Dickinson, S. F. Cheesman, J. F. Colyer, J. A Mallet, and E. Preedy. The garden party at Brompton, was well attended, and the grounds of the Cancer Hospital looked very bright and pretty. During the allotted time tea and other light refreshments were served to the company, as well as to those patients who were strong enough to avail themselves of the opportunity of appearing in the grounds, while the proceedings were enlivened by the performances of the excellent band of the A division of the Metropolitan Police.

